# A Lane Detection Method Based on a Ridge Detector and Regional G-RANSAC

**DOI:** 10.3390/s19184028

**Published:** 2019-09-18

**Authors:** Zefeng Lu, Ying Xu, Xin Shan, Licai Liu, Xingzheng Wang, Jianhao Shen

**Affiliations:** College of Mechatronics and Control Engineering, Shenzhen University, Shenzhen 518060, China; luzefeng_work@163.com (Z.L.); shanxin6812@126.com (X.S.); saiiasllc@163.com (L.L.); xingzheng.wang@szu.edu.cn (X.W.); shenjianhao@email.szu.edu.cn (J.S.)

**Keywords:** lane division lines’ detection, ridge detector, BP neural network, feature extraction, RANSAC

## Abstract

Lane detection plays an important role in improving autopilot’s safety. In this paper, a novel lane-division-lines detection method is proposed, which exhibits good performances in abnormal illumination and lane occlusion. It includes three major components: First, the captured image is converted to aerial view to make full use of parallel lanes’ characteristics. Second, a ridge detector is proposed to extract each lane’s feature points and remove noise points with an adaptable neural network (ANN). Last, the lane-division-lines are accurately fitted by an improved random sample consensus (RANSAC), termed the (regional) gaussian distribution random sample consensus (G-RANSAC). To test the performances of this novel lane detection method, we proposed a new index named the lane departure index (LDI) describing the departure degree between true lane and predicted lane. Experimental results verified the superior performances of the proposed method over others in different testing scenarios, respectively achieving 99.02%, 96.92%, 96.65% and 91.61% true-positive rates (TPR); and 66.16, 54.85, 55.98 and 52.61 LDIs in four different types of testing scenarios.

## 1. Introduction

Lane-division line detection plays a critical role in improving the safety level of intelligent electric vehicles (IEVs). Currently, there are two main methods to detect lane-division-lines: feature-based detection and model-based detection. Feature-based detection extracts the edge location distribution, connected shadow area, color and texture differences from graphs to detect lane-division-lines [1,2,3]. Zhang proposed a Hough transform based fitting-lane method for tracking [4]. Yoo used gradient-enhancing conversion for illumination-robust lane detection [5]. Geiger designed a Bayes model to discriminate lane-division line pixels from other pixels [6]. Peng proposed a lane-division line detection method with the statistical Hough transform based on a gradient constraint [7]. To extract significant features, Ma converted the color space of RGB to the CIELab color model and detected the lane-division-lines by using k-means clustering [8]. Son proposed a lane detection method based on the color feature and clustering method [9]. Jung proposed a lane-division line detection method based on the Haar feature [10]. Wang used lane detection by combining the self-clustering algorithm, fuzzy C-mean, and fuzzy rule to process the spatial information and Canny algorithm to extract edge features [11]. Other feature-based detection methods [12,13,14,15,16,17] also achieved good performances in normal-conditions scenarios; however, the main drawback of this approach is that it is easily disturbed by noise, as it ignores the model of the lane-division-lines.

Aiming to solve this shortcoming, many model-based detection methods are proposed. Zhou proposed a novel lane detection method based on the geometrical model and Gabor filter [18]. Wang proposed a lane detection approach based on inverse perspective mapping [19]. Baofeng proposed a detection system with a linear approximation method [20]. Although lane-division-lines detection technology has shown great development [21,22,23,24,25,26], feature extraction is still heavily influenced by complex road situations, such as light variance, under-road road signs and shadow interference [27]. Hence, more researchers are focusing on deep learning-based detection methods [28,29,30,31,32], which are showing better results, but these models heavily depend on the quality of training samples [33]. The artificial neural network is applied to remove noise from the image or feature map. Rashidha proposed an adaptive-size median filter for pulse noise removal based on a neural network [34]. With the development of computation, a deeper neural network was applied to reduce noise in samples. Kim Y proposed an adaptive filter based convolutional neural network [35]. Kuang removed noise by a deep convolution neural network, which showed good performances in various, unknown-noise scenarios [36].

In this paper, we aim to solve this problem by combining the advantages of feature detection and model detection methods. In particular, we propose a ridge detector to extract ridges from the aerial view map. Then, we remove the noise according to the lane-division-lines model. A sample set is generated by combining a ridge-positive sample and a noise-negative sample. In order to improve the robustness of the ridge detector, we fully utilize the information between frames. Specifically, we design a six-dimensional feature for each sample point to retrain a three-layer backpropagation (BP) neural network of each ten frames [37]. The six-dimensional feature consists of the abscissa; ordinate values; 3 × 3 convolution filter, 7 × 7 convolution filter and 11 × 11 convolution filter feature values; and the frequency within ten frames. We use the detection data set to update the neural network weights in real-time for the next ten frames’ detection. The confidence level for each of the ridge points is decided based on the results of ridge detector. The confidence values are decided by the corresponding pixels of the two-dimensional Gaussian distribution’s covariance matrix. To robustly fit the ridge points, we sample three ridge points from three different areas then generate *k* new points by two-dimensional Gaussian inverse-transformation sampling for each selected ridge point. Last, we fit the 3*k* + 3 points by the random sample consensus (RANSAC) algorithm, in which the least-squares’ objective function is solved by the stochastic sub-gradient descent (SGD). In this research, we used an adaptable neural network (ANN) to discriminate noise and ridge points, and improved the traditional RANSAC algorithm by considering the confidence of remaining ridge-feature points.

The remainder of this paper is organized as follows. In Section 2, related work is introduced. In Section 3, the lane-division line feature extraction method, based on an adaptable ridge detector and the between-frames neural network, is proposed, and the regional gaussian distribution random sample consensus (G-RANSAC) fitting method is proposed as well. The experimental results are shown in Section 4, and finally, the conclusion is drawn in Section 5.

*Nomenclature*: Let I stand for real matrix of an image; $I^{i,j}$ stands for the row i column j element of matrix I. h and w represent for height and width of an image, respectively. Symbol ⊗ represents for convolution operation; min(I) and max(I) represent the minimum and maximum element of matrix I, respectively.

## 2. Related Work

Lane-division lines can be detected using a static camera sensor or vehicle-mounted camera sensor. For the static camera sensor, the application scenarios include precise vehicle positioning and intelligent transportation. The main methods include method are based on the trajectories of vehicles [38,39] and pixel-entropy [40]. For the vehicle-mounted camera sensor, the application scenarios include intelligent vehicles and advanced driver assistant systems (ADAS) [41,42]. In this paper, we focus on lane-division line detection based on vehicle-mounted camera sensor.

Lane detection methods based on a moving camera sensor are divided into two steps, lane feature extraction, and feature points fitting. For feature extraction, Sobel and Canny edge features are usually used as the lane division lane feature, but the edge feature is susceptible to noise interference. To solve this problem, a ridge detected method based on ridge-feature is proposed [43], which contains two matrix-convolution operations, two matrix-differential operations, three matrix-point product operations, and a divergence calculation. In this paper, we propose a simpler ridge detection method based on lane width with better results in real time, as shown in Section 4.1.

For feature point fitting, Hough transform is the usual method [15], but it is an inefficient method, thus J Guo proposed a lane-division, lane-feature-fitting method based on RANSAC [44]. The main steps of this method are the following: (1) Fewer fitting points are selected randomly from the whole point set. (2) The model is fit based on the selected points in step 1 by the least-square method. (3) Step 1 is repeated until the maximum iterative number is reached. (4) The best fitting model from multiple iterations is selected, according to whole point set’s lowest error. Compared with Hough transform, this method improves fitting efficiency. However, randomly selecting fitting points from whole feature points is a bad strategy for lane detection; the reason is illustrated in Figure 9b. Therefore, in Section 3.4 of this paper, we propose to divide the ridge-feature map into three regions, and then resample, based on the Gaussian distribution according to ridge coefficient. The experimental results show significantly improved fitting efficiency.

## 3. Materials and Methods

**Figure 1:**
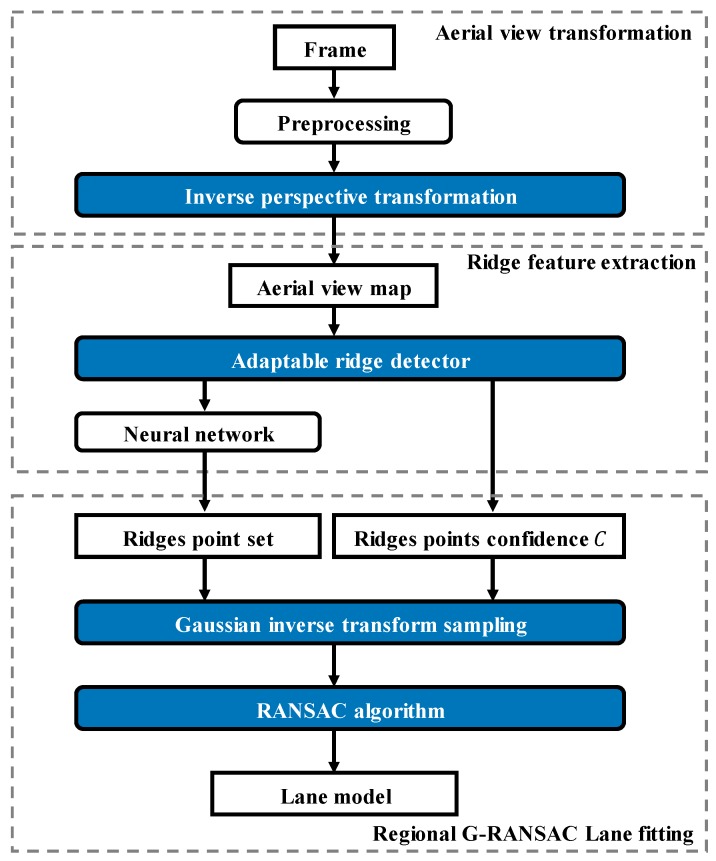
Detection algorithm block diagram.

The detection algorithm is proposed here, which is divided into five sub-sections, as shown in Figure 1. In Section 3.1, a graphical preprocessing method based on inverse perspective transformation is presented to obtain the aerial view map. In Section 3.2, a feature extraction method based on an adaptable ridge detector is developed to extract the feature of ridges in the lane-division-lines. In Section 3.3, we extract a six-dimensional feature for each pixel to attain the neural network, which is used to discriminate between noise and ridge points. In Section 3.4, the regional G-RANSAC is proposed to robustly fit the lane-division-lines. In Section 3.5, we propose a new index, named the lane departure index (LDI), to test the performances of this lane detection method.

### 3.1. A Graphical-Preprocessing-Method Based Inverse-Perspective Transformation

**Figure 2:**
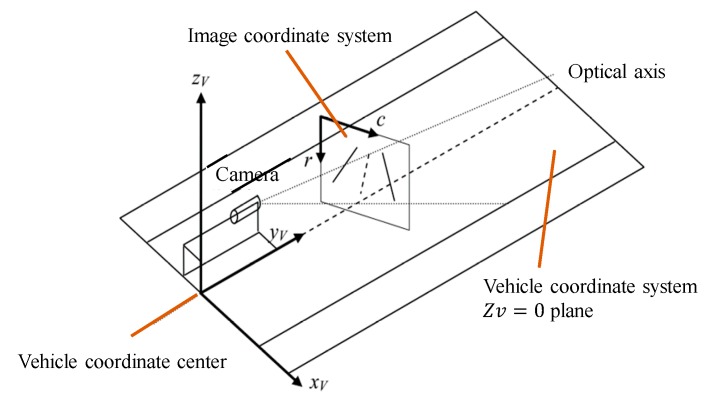
The principles of the inverse perspective transformation.

As the frame captured from the camera is an RGB-image with noise, preprocessing methods consist of a gray image transformation and using a 5 × 5 median filter to reduce image noise. However, there are many additional interferences from image background information. Therefore, we select the region of interest (ROI) by inverse perspective transformation to attain the aerial view map, which eliminates perspective-side effects. According to the lane-division line standard [43], we choose a 35 m by 11.5 m region in the Cartesian coordinate system. The principle of the inverse perspective transformation is shown in Figure 2; $\left(x_{v},y_{v},z_{v}\right)$ represents the vehicle coordinate system and (c,r) represents the image coordinate system. In the inverse perspective transformation, we convert the image coordinate system (c,r) into a real-world, three-dimensional coordinate system $\left(x_{v},y_{v},0\right)$. The transforming relationship is described as follows:

(1) $$\{\begin{array}{c} x_{v}=hcTG\left(\frac{2\alpha }{s_{x}-1}r-\alpha +\theta \right)\sin\left(\frac{2\alpha }{s_{x}-1}c-\alpha +\gamma \right)+d\\ y_{v}=hcTG\left(\frac{2\alpha }{s_{y}-1}r-\alpha +\theta \right)\cos\left(\frac{2\alpha }{s_{y}-1}c-\alpha +\gamma \right)+l \end{array}\;c\in N^{+},\;r\in N^{+},0<c\leq s_{x},0<r\leq w-s_{y}$$

where (d,l,h) represent the camera’s center coordinates in the vehicle coordinate system; and T and G denote the transfer and rotation matrices, respectively. $s_{x}$ and $s_{y}$ stand for the image resolution; θ is the angle between optical axis and the $z_{v}$ = 0 plane; γ is the angle between optical axis and the vehicle coordinate system’s axes, $y_{v}$; α denotes half of the camera view angle.

We convert the image to an aerial view map with Equation (1).

### 3.2. Ridge Detector

**Figure 3:**
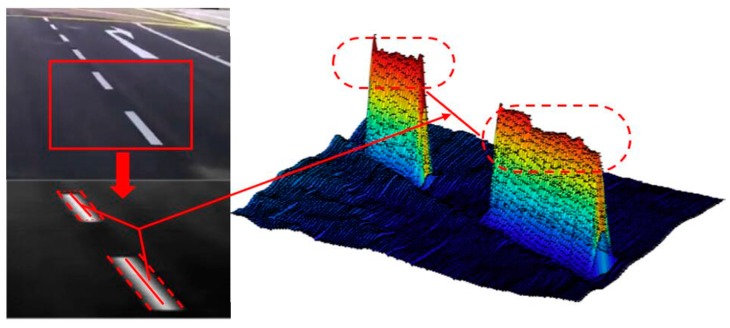
Ridge-feature illustration.

In general, ridges are low-level features in gray images. In comparison with edge features, ridge-features are more suitable to describe lane-division-lines in situations such as vehicle shadow interference, worn-out ground signs and insufficient illumination [19]. As shown in Figure 3, the ridge is the center line. In the traditional ridge detected method in Section 2, the gradient vector calculation and multiple matrix operation require high computational cost.

**Figure 4:**
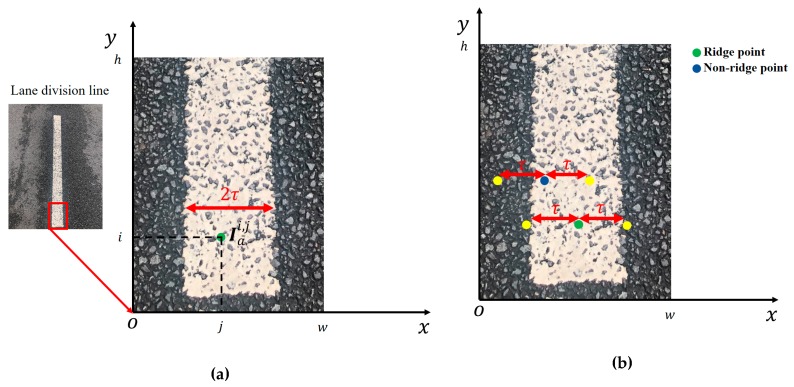
Ridge detector illustration. (**a**) ridge point illustration. (**b**) Equation (2) illustration.

According to the geometry of lane-division-lines in the aerial view map $I_{a}$, we proposed a simple ridge detector based on lane width. As shown in Figure 4a, we define the green point $I_{a}^{i,j}$ as row i column j pixel’s value, in the image coordinate system. Row i denotes ordinate and column j denotes abscissa; we define 2τ as the lane-division line transverse pixel number in the aerial view map. $I_{r}$ stands for ridge-feature map, which is defined by Equation (2).

(2) $$I_{r}^{i,j}=\left(I_{a}^{i,j}-I_{a}^{i,j-\tau }\right)+\left(I_{a}^{i,j}-I_{a}^{i,j+\tau }\right)\;i\in N^{+},\;j\in N^{+},0<i\leq h,\;\tau <j\leq w-\tau$$

where τ denotes half number of lane-division line transverse pixels.

As shown in Figure 4b, $v_{in}$ is defined as the pixel value of the point in a lane-division line and $v_{out}$ is defined as the pixel value of the point outside a lane-division line, where $v_{in}>v_{out}$. The green point $I_{a}^{i,j}$ represents the ridge point value, $v_{in}$; its left and bright yellow point are outside the lane-division line with a $v_{out}$ value. The blue point represents non-ridge point with a $v_{in}$ value, its left yellow point is outside the lane-division line with a $v_{out}$ value, and a bright yellow point is in a lane-division line with a $v_{in}$ value. The term $\left(I_{a}^{i,j}-I_{a}^{i,j+\tau }\right)$ of a green point is larger than the term $\left(I_{a}^{i,j}-I_{a}^{i,j+\tau }\right)$ of a blue point, and the term $\left(I_{a}^{i,j}-I_{a}^{i,j-\tau }\right)$ of a green point equivalent to the term $\left(I_{a}^{i,j}-I_{a}^{i,j-\tau }\right)$ of a blue point. According to Equation (2), the ridge-feature value $I_{r}^{i,j}$ of a green point is larger than the ridge-feature value $I_{r}^{i,j}$ of a blue point.

In order to increase the difference between the $I_{r}^{i,j}$ of a ridge point and a non-ridge point, on the basis of Equation (2), we add a punishment term $\left|I_{a}^{i,j+\tau }-I_{a}^{i,j-\tau }\right|$, as shown in Equation (3). At the ridge point, the punishment term equates to zero, but the term of the non-ridge point is larger than zero.

(3) $$I_{r}^{i,j}=\left(I_{a}^{i,j}-I_{a}^{i,j-\tau }\right)+\left(I_{a}^{i,j}-I_{a}^{i,j+\tau }\right)-\left|I_{a}^{i,j+\tau }-I_{a}^{i,j-\tau }\right|$$

We simplify Equation (3) to obtain the following ridge detector mathematical model:

(4) $$I_{r}^{i,j}=\left(-I_{a}^{i,j-\tau }+2I_{a}^{i,j}-I_{a}^{i,j+\tau }\right)-\left|I_{a}^{i,j+\tau }-I_{a}^{i,j-\tau }\right|$$

According to Equation (4), we calculate the row i column j ridge-feature value $I_{r}^{i,j}$ for each pixel $I_{a}^{i,j},\;0<i\leq h,\;\tau <j\leq w-\tau$, which is shown in matrix form:

(5) $$\begin{array}{ll} \left[\begin{array}{ccc} I_{r}^{h,1+\tau } & \cdots & I_{r}^{h,w-\tau }\\ \vdots & \ddots & \vdots \\ I_{r}^{1,1+\tau } & \cdots & I_{r}^{1,w-\tau } \end{array}\right] & =[\begin{array}{ccc} \left(-I_{a}^{h,1}+2I_{a}^{h,1+\tau }-I_{a}^{h,1+2\tau }\right)-\left|I_{a}^{h,1+2\tau }-I_{a}^{h,1}\right| & \cdots & \left(-I_{a}^{h,w-2\tau }+2I_{a}^{h,w-\tau }-I_{a}^{h,w}\right)-\left|I_{a}^{h,w}-I_{a}^{h,w-2\tau }\right|\\ \vdots & \ddots & \vdots \\ \left(-I_{a}^{1,1}+2I_{a}^{1,1+\tau }-I_{a}^{1,1+2\tau }\right)-\left|I_{a}^{1,1+2\tau }-I_{a}^{1,1}\right| & \cdots & \left(-I_{a}^{1,w-2\tau }+2I_{a}^{1,w-\tau }-I_{a}^{1,w}\right)-\left|I_{a}^{1,w}-I_{a}^{1,w-2\tau }\right| \end{array}]\\ & =[\begin{array}{ccc} \left(-I_{a}^{h,1}+2I_{a}^{h,1+\tau }-I_{a}^{h,1+2\tau }\right) & \cdots & \left(-I_{a}^{h,w-2\tau }+2I_{a}^{h,w-\tau }-I_{a}^{h,w}\right)\\ \vdots & \ddots & \vdots \\ \left(-I_{a}^{1,1}+2I_{a}^{1,1+\tau }-I_{a}^{1,1+2\tau }\right) & \cdots & \left(-I_{a}^{1,w-2\tau }+2I_{a}^{1,w-\tau }-I_{a}^{1,w}\right) \end{array}]-[\begin{array}{ccc} \left|I_{a}^{h,1+2\tau }-I_{a}^{h,1}\right| & \cdots & \left|I_{a}^{h,w}-I_{a}^{h,w-2\tau }\right|\\ \vdots & \ddots & \vdots \\ \left|I_{a}^{1,1+2\tau }-I_{a}^{1,1}\right| & \cdots & \left|I_{a}^{1,w}-I_{a}^{1,w-2\tau }\right| \end{array}] \end{array}$$

We redefine ridge-feature map $I_{r}$:

(6) $$I_{r}=\left[\begin{array}{ccc} I_{r}^{h,1+\tau } & \cdots & I_{r}^{h,w-\tau }\\ \vdots & \ddots & \vdots \\ I_{r}^{1,1+\tau } & \cdots & I_{r}^{1,w-\tau } \end{array}\right],$$

aerial view map $I_{a}$:

(7) $$I_{a}=\left[\begin{array}{ccc} I_{a}^{h,1} & \cdots & I_{a}^{h,w}\\ \vdots & \ddots & \vdots \\ I_{a}^{1,1} & \cdots & I_{a}^{1,w} \end{array}\right]$$

and one-dimensional ridge filters R and P:

(8) $$R=\left[-1,\underset{\tau -1\;zeros}{\underset{︸}{0,\ldots ,0}},2,\underset{\tau -1\;zeros}{\underset{︸}{0,\ldots ,0}},-1\right]$$

(9) $$P=[1,\underset{2\tau -1\;zeros}{\underset{︸}{0,\ldots ,0}},-1]$$

where τ denotes half the number of the lane-division line’s transverse pixels.

According to Equations (6)–(9), we simplify Equation (5):

(10) $$I_{r}=R⊗I_{a}-\left|P⊗I_{a}\right|$$

According to Equation (10), we can convert the aerial view map $I_{a}$ to ridge-feature map $I_{r}$.

We obtain the normalized ridge-feature map $I_{n}$ by Equation (11).

(11) $$I_{n}=\left[\begin{array}{ccc} \frac{I_{r}^{h,1+\tau }-min\left(I_{r}\right)}{max\left(I_{r}\right)-min\left(I_{r}\right)} & \cdots & \frac{I_{r}^{h,w-\tau }-min\left(I_{r}\right)}{max\left(I_{r}\right)-min\left(I_{r}\right)}\\ \vdots & \ddots & \vdots \\ \frac{I_{r}^{1,1+\tau }-min\left(I_{r}\right)}{max\left(I_{r}\right)-min\left(I_{r}\right)} & \cdots & \frac{I_{r}^{1,w-\tau }-min\left(I_{r}\right)}{max\left(I_{r}\right)-min\left(I_{r}\right)} \end{array}\right]$$

**Figure 5:**
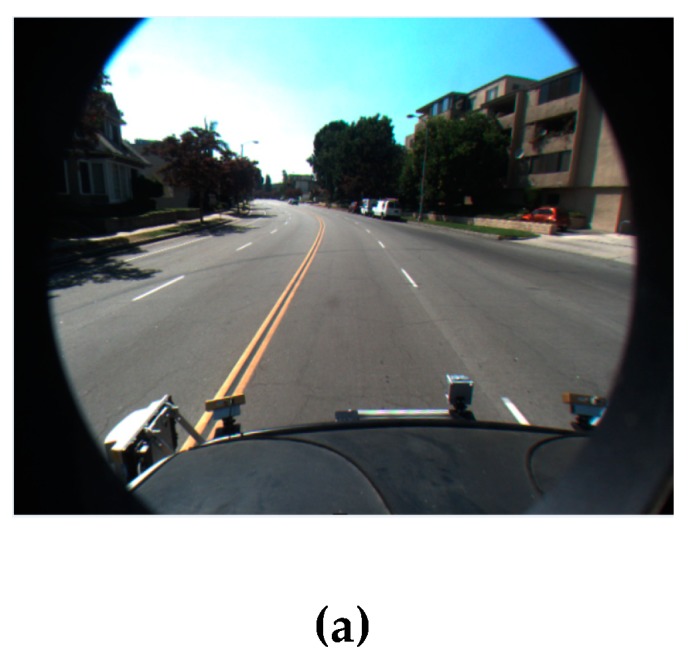

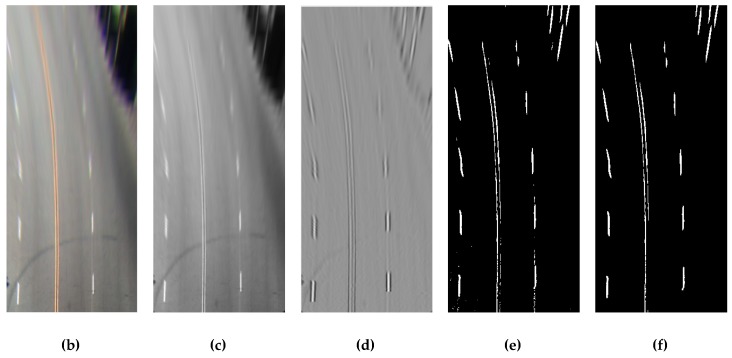
Ridge-feature extraction illustration. (**a**) Original map I. (**b**) Aerial view map $I_{a}$. (**c**) Gray map. (**d**) Ridge map $I_{r}$. (**e**) Ridge binary map $I_{b}$. (**f**) Map when areas with pixel numbers less than 30 are removed.

Pictorial examples of ridge-feature extraction are shown in Figure 5.

$I_{n}$ describes the possibility of a pixel belonging to a ridge point set; we set the value of $I_{n}$ as the confidence level for the regional G-RANSAC algorithm in Section 3.4.

Thus, we attain the statistical histogram of $I_{n}$ and select the highest bin corresponding value as the threshold t to covert $I_{n}$ to a binary ridge binary image $I_{b}$. According to the experimental results, the mathematical morphology process is applied to image $I_{b}$ to remove areas with pixel numbers less than 30.

### 3.3. 6-Dimensional Feature Extraction and Retraining a BP Neural Network for Removing Noise

In this section, we propose an adaptable classification method for binary ridge-feature image $I_{b}$ to discriminate noise and ridge points. The noise point is comprised of another objection noise point and imaging noise point. No-objection noise is caused by the sign on the ground, tree shadow, or other vehicles; therefore, they appear in particular areas. We remove the no-objection noise point by pixel position information according to the lane-division line model. The imaging noise points are randomly distributed and caused by the camera sensor, but it is a separate and accidental process. Therefore, we remove the imaging noise points by calculating the number of surrounding feature points and the frequency of 10 consecutive frames. The frequency affects the real-time performance and adaptability of neural network. The frequency of 10 was selected based on experimental results.

A six-dimensional feature $F^{i,j}$ for the row i column j pixel is proposed to discriminate noise and ridge points, which is as follows:

(12) $$F^{i,j}=\left[i,\;j,N_{3}^{i,j},N_{7}^{i,j},N_{11}^{i,j},k_{ij}\right]\;N_{3}=ones\left(3\right)⊗I_{b}\;N_{7}=ones\left(7\right)⊗I_{b}\;N_{11}=ones\left(11\right)⊗I_{b}$$

where $I_{b}$ stands for binary ridge-feature image; i, j denotes row i column j; $N_{3}$, $N_{7}$ and $N_{11}$ denote the convolution feature matrices; ones(n) is a n-rank square matrix’s element equal to 1; $N_{3}^{i,j}$ is the row i column j element of the convolution feature matrix $N_{3}$; $N_{7}^{i,j}$ is the row i column j element of convolution feature matrix $N_{7}$; $N_{11}^{i,j}$ is the row i column j element of convolution feature matrix $N_{11}$; and $k_{ij}$ represents for the frequency of pixel (i, j) within 10 frames.

**Figure 6:**
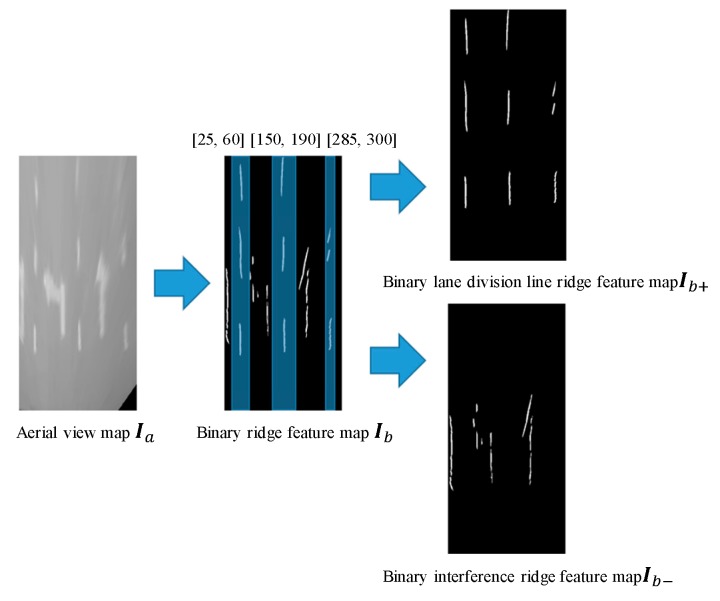
Pictorial example for dividing binary ridge map $I_{b}$.

According to the lane-division line model, binary ridge image $I_{b}$ can be divided into two sets. The ridge point set is expressed as $I_{b+}$, in which abscissa i∈=(25,60) or (150,190) or (285,300), which came from experiment result in Figure 6. However, the constant bounds are ineffective when in the vehicle change lane. Variable bounds are an effective method to handle this scenario, which will be exploited in the feature. The noise point set is expressed as $I_{b-}$. Point set $I_{b+}$ would be processed by a well-trained BP neural network and further divided into ridge point subset $I_{b++}$ and no-ridge point subset $I_{b+-}$. The ridges’ subset $I_{b++}$ is be fitted to the line.

**Figure 7:**
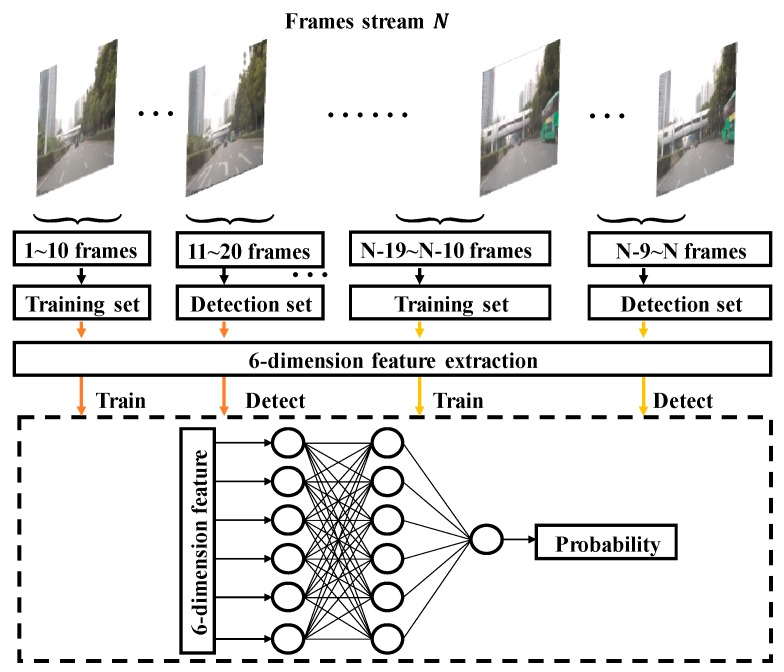
Retrain the backpropagation (BP) neural network for each 10 frames.

To improve the detection robustness, within each 10 frames, we retrain the BP neural network by a positive sample set $I_{b++}$ and negative sample sets $I_{b+-}$ and $I_{b-}$. Cross-entropy is applied as a loss function, and the sigmoid function is adopted as an activation function. The structure of the BP neural network is shown in Figure 7.

### 3.4. Regional G-RANSAC

**Figure 8:**
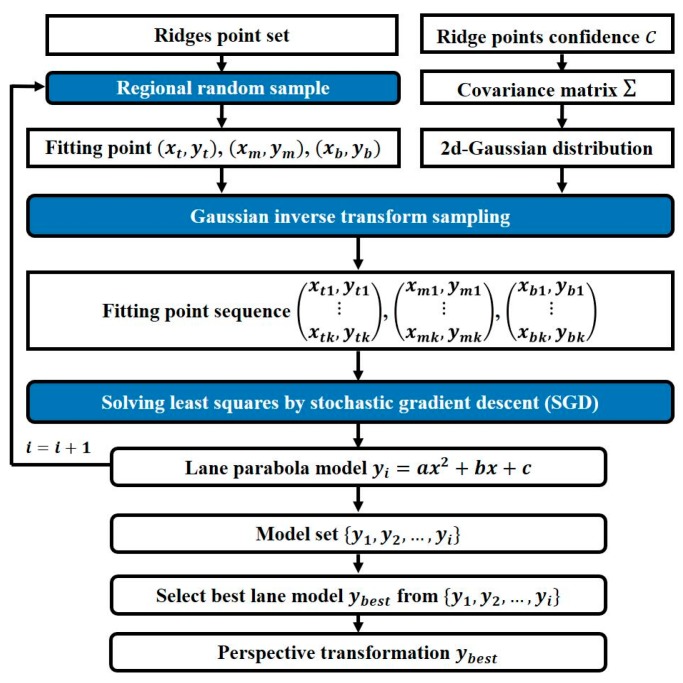
Regional gaussian distribution random sample consensus (G-RANSAC) land fitting block diagram.

**Figure 9:**
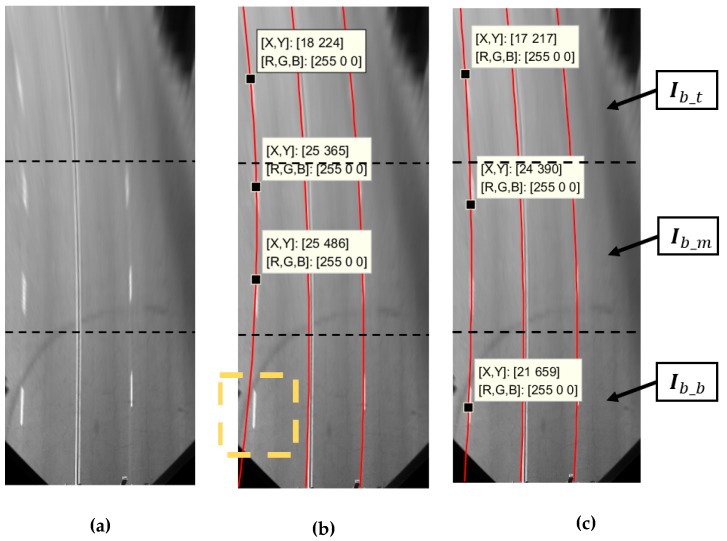
Regional fitting points selection illustration. (**a**) Aerial map. (**b**) Select. fitting points randomly from whole map. (**c**) Select fitting points randomly from three areas.

The RANSAC algorithm is applied to fit the point set, which contains a number of noise points [43]. However, random sampling from the whole point set is not a good strategy for lane model fitting. As shown in Figure 8, we improve RANSAC algorithm by considering ridge point confidence and sampling areas. The binary ridges feature map $I_{b}$ is divided into three areas $I_{b\_t}$, $I_{b\_m}$ and $I_{b\_b}$, shown in Figure 9a. We randomly select a ridge point from each area, defined as $\left(x_{t},y_{t}\right)$, $\left(x_{m},y_{m}\right)$, and $\left(x_{b},y_{b}\right)$, respectively. Fitting efficiency would be improved in Figure 9c, compared with Figure 9b.

The feature points selection method is suitable for lane fitting, but it is still possible to select noise points. Therefore, we assume that the selected row, i column, j ridge point and $I_{b}^{i,j}$ coordinate (j,i), is subject to two-dimensional Gaussian distribution, in which probability density function is defined as follows:

(13) $$f_{j,i}\left(x,y\right)=\frac{1}{2\pi \sqrt{\left(\sigma_{1}^{2}\sigma_{2}^{2}\right)}}\exp\left(-\frac{1}{2}\left[{\left(\frac{x-u_{1}}{\sigma_{1}}\right)}^{2}+{\left(\frac{y-u_{2}}{\sigma_{2}}\right)}^{2}\right]\right)\;x\in N^{+},\;y\in N^{+},0<x\leq w,\;0<y\leq h$$

where $\left(\begin{array}{c} u_{1}\\ u_{2} \end{array}\right)=\left(\begin{array}{c} j\\ i \end{array}\right)$ means vector, and $\sigma_{1}$ and $\sigma_{2}$ are the covariance matrix $\Sigma_{i,j}$’s elements. We hypothesis that variables x and y are non-correlated, hence covariance matrix $\Sigma_{i,j}$ is defined as $\Sigma_{i,j}=\left[\begin{array}{cc} \sigma_{1}{}^{2} & 0\\ 0 & \sigma_{2}{}^{2} \end{array}\right]$.

The covariance matrix $\Sigma_{i,j}$ describes the uncertainty degree of selected point $I_{b}^{i,j}$ belonging to the ridge point set. In other words, the selected point $I_{b}^{i,j}$ in the ridge point set is the fitting target, so we set the covariance matrix $\Sigma_{i,j}$ as a small value to limit the inverse transformation resampling range. The other case is that the selected point $I_{b}^{i,j}$ is not in the ridge point set; then, we set a big covariance matrix $\Sigma_{i,j}$, giving a chance for this point to generate ridge points at the inverse-transformation sampling stage.

We then define the confidence level $C^{i,j}$ as the probability of selected point $I_{b}^{i,j}$, which belongs the to ridge point set. $C^{i,j}$ denotes the row i, column j element of the ridge points’ confidence matrix C, which is defined in Equation (14):

(14) $$C=\frac{ones\left(3\right)⊗I_{n}}{9}$$

where ones(3) is when the three-rank square matrix’s element equals 1. $I_{n}$ stands for normalized ridge-feature map. Covariance matrix $\Sigma_{i,j}$ is calculated with confidence $C^{i,j}$ of the point $I_{b}^{i,j}$ in binary ridge-feature map $I_{b}$, as shown in Equation (15):

(15) $$\Sigma_{i,j}=\left[\begin{array}{cc} \frac{\alpha }{C^{i,j}} & 0\\ 0 & \frac{\alpha }{C^{i,j}} \end{array}\right]$$

where α denotes scale coefficient.

In summary, the selected point $I_{b}^{i,j}$ initiates a two-dimensional Gaussian distribution with mean vector (j,i) and covariance matrix $\Sigma_{i,j}$. Because abscissa j and ordinate i are non-correlated, the two-dimensional Gaussian distribution can be converted into two one-dimensional Gaussian distributions, $N\left(\mu ,\sigma^{2}\right)$. Therefore, abscissa distribution $x\sim N\left(j,\frac{\alpha }{C^{i,j}}\right)$ and ordinate distribution $y\sim N\left(i,\frac{\alpha }{C^{i,j}}\right)$ arise; their probability density functions are shown in Equations (16) and (17), respectively.

(16) $$f\left(x\right)=\frac{1}{\sqrt{2\pi \times \frac{\alpha }{C^{i,j}}}}exp\left(-\frac{{\left(x-j\right)}^{2}}{2\frac{\alpha }{C^{i,j}}}\right)$$

(17) $$f\left(y\right)=\frac{1}{\sqrt{2\pi \times \frac{\alpha }{C^{i,j}}}}exp\left(-\frac{{\left(y-i\right)}^{2}}{2\frac{\alpha }{C^{i,j}}}\right)$$

The corresponding cumulative distribution function is shown in Equations (18) and (19).

(18) $$F\left(x\right)=\frac{1}{\sqrt{2\pi \times \frac{\alpha }{C^{i,j}}}}\int_{-\infty }^{x}exp\left(-\frac{{\left(x-j\right)}^{2}}{2\frac{\alpha }{C^{i,j}}}\right)dx$$

(19) $$F\left(y\right)=\frac{1}{\sqrt{2\pi \times \frac{\alpha }{C^{i,j}}}}\int_{-\infty }^{y}exp\left(-\frac{{\left(y-i\right)}^{2}}{2\frac{\alpha }{C^{i,j}}}\right)dy$$

where exp(x) denotes an exponential function, α denotes a scale coefficient and (j,i) is the coordinate point of ridge-feature map $I_{r}$. In Equations (18) and (19), (j,i) represents the mean vector of the Gaussian distribution.

We describe Equations (18) and (19) by the error function $erf\left(x\right)=\frac{2}{\sqrt{\pi }}\int_{0}^{x}e^{-\eta^{2}}d\eta$, as shown in Equations (20) and (21), respectively.

(20) $$\Phi_{x}\left(z\right)=\frac{1}{2}\left[1+erf\left(\frac{z-j}{\sqrt{2\frac{\alpha }{C^{i,j}}}}\right)\right]\;z\in N^{+},0<z\leq w$$

(21) $$\Phi_{y}\left(t\right)=\frac{1}{2}\left[1+erf\left(\frac{t-i}{\sqrt{2\frac{\alpha }{C^{i,j}}}}\right)\right]\;t\in N^{+},0<t\leq h$$

The inverse function of $\Phi_{x}\left(z\right)$ and $\Phi_{y}\left(t\right)$ is shown in Equations (22) and (23).

(22) $$\Phi_{x}{}^{-1}\left(p\right)=\sqrt{2\frac{\alpha }{C^{i,j}}}\times erf^{-1}\left(2p-1\right)+j\;min\left(\Phi_{x}\left(z\right)\right)\leq p\leq max\left(\Phi_{x}\left(z\right)\right)$$

(23) $$\Phi_{y}{}^{-1}\left(q\right)=\sqrt{2\frac{\alpha }{C^{i,j}}}\times erf^{-1}\left(2q-1\right)+i\;min\left(\Phi_{y}\left(t\right)\right)\leq q\leq max\left(\Phi_{y}\left(t\right)\right)$$

where α denotes scale coefficient, and (j,i) is a coordinate point of ridge-feature map $I_{r}$, in Equations (18) and (19). (j,i) denotes the mean vector of the Gaussian distribution.

According to inverse transformation sampling [45], we attain the selected point sequence $\left(\begin{array}{c} x_{i1},y_{i1}\\ \vdots \\ x_{ik},y_{ik} \end{array}\right)$ by $\left(\begin{array}{c} \Phi_{x}{}^{-1}\left(p_{1}\right),\Phi_{y}{}^{-1}\left(q_{1}\right)\\ \vdots \\ \Phi_{x}{}^{-1}\left(p_{k}\right),\Phi_{y}{}^{-1}\left(q_{k}\right) \end{array}\right)$, where k represents the inverse transformation sampling numbers. $\left(p_{1},p_{2},\;\ldots ,p_{k}\right)$ and $\left(q_{1},q_{2},\;\ldots ,q_{k}\right)$ are subject to uniform distribution $\left(\begin{array}{c} x_{i1}\\ \vdots \\ x_{ik} \end{array}\right)$, which is subject to $N\left(j,\frac{\alpha }{C^{i,j}}\right)$ and $\left(\begin{array}{c} y_{i1}\\ \vdots \\ y_{ik} \end{array}\right)$, which are subject to $N\left(i,\frac{\alpha }{C^{i,j}}\right)$.

The traditional RANSAC algorithm, which randomly fits selected points by least-squares, in which the objective function is l, is shown in Equation (24).

(24) $$min\;l=\sum_{i=1}^{n}{\left(y_{i}-f\left(x_{i}\right)\right)}^{2}$$

where n represents the number of fitting points, $\left(x_{i},y_{i}\right)$ denotes the ith fitting points and $f\left(x_{i}\right)$ denotes the fitting model. We define $f\left(x\right)=ax^{2}+bx+c$, for each $x_{i}$, where $x_{i}$ belongs to (0, w]. In this paper, we choose the stochastic sub-gradient descent (SGD) method [46] to solve Equation (24), because the number of selected point sequence $\left(\begin{array}{c} x_{i1},y_{i1}\\ \vdots \\ x_{ik},y_{ik} \end{array}\right)$ is large if the inverse-transform sampling parameter k is large. The objective function’s partial derivatives with respect to a, b and c are as follows:

(25) $$\frac{\partial L}{\partial a}=2\sum_{i=1}^{n}\sum_{j=1}^{k}\left(ax_{ij}^{2}+bx_{ij}+c-y_{ij}\right)x_{ij}^{2}$$

(26) $$\frac{\partial L}{\partial b}=2\sum_{i=1}^{n}\sum_{j=1}^{k}\left(ax_{ij}^{2}+bx_{ij}+c-y_{ij}\right)x_{ij}$$

(27) $$\frac{\partial L}{\partial c}=2\sum_{i=1}^{n}\sum_{j=1}^{k}\left(ax_{ij}^{2}+bx_{ij}+c-y_{ij}\right)$$

where n represents the number of randomly selected points and k represents the number of inverse transform sampling.

The stochastic sub-gradient descent for solving the least-squares receives four input parameters: (i) step size λ, (ii) the number of iterations $t_{max}$, (iii) the number of examples to use for calculating sub-gradient l, (iv) and the fitting point sequence X. Algorithm 1 describes the proposed method in pseudocode.

**Algorithm 1** The stochastic sub-gradient descent for solving least-squares1: **Input:**
$\lambda ,\;t_{max},\;l,\;X$ 2: **Initialize:**
$a^{\left(1\right)}=1$, $b^{\left(1\right)}=1$, $c^{\left(1\right)}=1$; 3: **For** t = 1, 2, …, $t_{max}$
**do** 4: Choose $X_{t}\subseteq X$, where $\left|X_{t}\right|=l$ 5: Set $\eta_{t}=\frac{1}{\lambda t}$ 6: $a^{\left(t+1\right)}\leftarrow a^{\left(t\right)}-\eta_{t}\frac{\partial L}{\partial a}$, $b^{\left(t+1\right)}\leftarrow b^{\left(t\right)}-\eta_{t}\frac{\partial L}{\partial b}$, $c^{\left(t+1\right)}\leftarrow c^{\left(t\right)}-\eta_{t}\frac{\partial L}{\partial c}$ 7: **End for.**

### 3.5. Lane Departure Index (LDI)

The true-positive rate (TPR) and false-positive rate (FPR) are common indices in lane detection [16], which is used to measure the ratio of correctly fitting frames to the total frames, described as follows:

(28) $$\mathrm{TPR}=\frac{NTP}{NLT}$$

(29) $$\mathrm{FPR}=\frac{NFP}{NLT}$$

where NTP (number of true-positive) is the number of correctly predicted lane detections; NLT (number of lanes positive) is the real lane number in the test video; and NFP (number of false-positive) is the number of wrongly predicted lane detections.

However, for a lane-division line, the judgment of TPR and FPR indices are binary: correct predictions or wrong predictions. It is very dangerous for an autopilot system to predict a lane division lane inaccurately, so it is necessary to find an index to describe the departure degree between the predicted lane and real predicted in a single frame.

**Figure 10:**
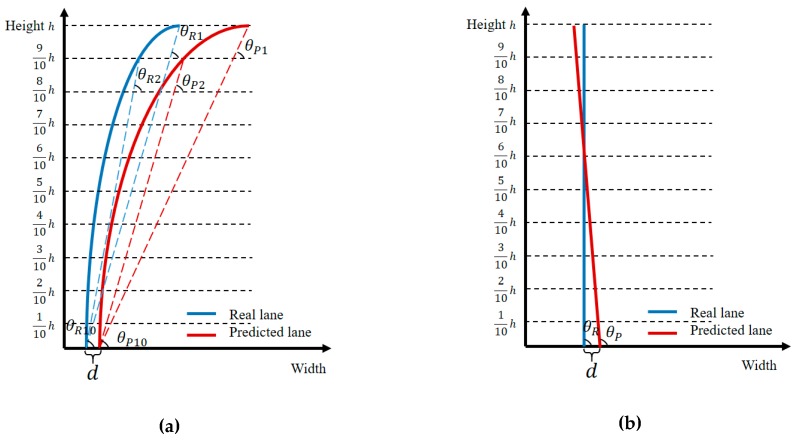
Illustration of lane departure index (LDI). (**a**) Curved lane in an aerial view map. (**b**) Straight lane in aerial view map.

Herein, we propose a new measure index termed the lane departure index (LDI) to describe the departure degree. The curve-line lane model is simplified to ten straight lines based on ten points of curved lane, as shown in Figure 10a and defined in Equation (30).

(30) $$\mathrm{LDI}=100-\sum_{j=1}^{m}\sum_{i=1}^{n}\left(1+\frac{d_{ji}}{w}\right)\times \left[\alpha_{1}{\left(\theta_{R1\_ji}-\theta_{P1\_ji}\right)}^{2}+\alpha_{2}{\left(\theta_{R2\_ji}-\theta_{P2\_ji}\right)}^{2}+\cdots +\alpha_{10}{\left(\theta_{R10\_ji}-\theta_{P10\_ji}\right)}^{2}\right]\;i\in N^{+},\;j\in N^{+},0<i\leq n,\;0<j\leq m,\;\alpha_{l}=\frac{1}{10},\;l\in N^{+},\;0<l\leq 10$$

where $d_{ji}$ denotes the abscissa error between ith real lane, and ith predicted lane of jth frame when the ordinate is zero; $\alpha_{1}$ to $\alpha_{10}$ are the weight of ten simplified straight line; $\theta_{R1\_ji}$ denotes the first simplified straight line of ith real lane in jth frame; $\theta_{P1\_ji}$ denotes the first simplified straight line of ith predicted lane in jth frame; $\theta_{R2\_ji}$ denotes the second simplified straight line of ith real lane in jth frame; $\theta_{P2\_ji}$ denotes the second simplified straight line of ith predicted lane in jth frame; $\theta_{R10\_ji}$ denotes the tenth simplified straight line of ith real lane in jth frame; $\theta_{P10\_ji}$ denotes the tenth simplified straight line of ith predicted lane in jth frame; m denotes video frame number; and n denotes the number of the jth frame real lane-division line.

Shown in Figure 10b, as a particular case of a two-degree polynomial curve lane, Equation (30) also holds true for a straight lane, and the terms $\left(\theta_{ji}^{R1}-\theta_{ji}^{P1}\right),\;\left(\theta_{ji}^{R2}-\theta_{ji}^{P2}\right),\;\cdots ,\;\mathrm{and}\;\left(\theta_{ji}^{R10}-\theta_{ji}^{P10}\right)$ are equal to each other.

## 4. Results

In this section, three experiments are carried out. In Section 4.1, there is a comparison experiment about the operating speed of the ridge-feature model [43]. The proposed method is given and the computational complexity is analyzed. In Section 4.2, the median filter, regional noise removing method and BP neural network are applied to remove noise adaptability, and their effectiveness is verified. In Section 4.3, the effectiveness of fitting method regional G-RANSAC is verified by comparing with traditional RANSAC and the Hough transform. Lastly, in Section 4.4, the comparative experimental results of the whole proposed method and other lane detection methods are given to verify the improvement in challenging scenarios.

### 4.1. An Analysis of the Ridge-Feature Extraction Method’s Operating Speed

The traditional ridge-feature extraction method [43] contains two matrix convolution operations, two matrix differential operations, three matrix point product operations and a divergence calculation. The proposed method in Equation (10) contains two matrix convolution operations and a matrix subtraction operation.

We compared the running speed of the traditional ridge-feature extraction method and the proposed method. Software platform: MATLAB R2018b. Hardware platform: CPU: Intel Core i5-4570 CPU (3.20 GHz), Memory: 32 GB and GPU: NVIDIA GeForce GTX 1080 Ti. Testing video: shown in Table 3.

As shown in Table 1, compared with method [43], the proposed method operating speed is improved 2.56, 2.70, 2.55 and 2.50 times in the four testing scenario videos respectively.

### 4.2. A BP Neural Network Applied to Remove Noise

**Figure 11:**
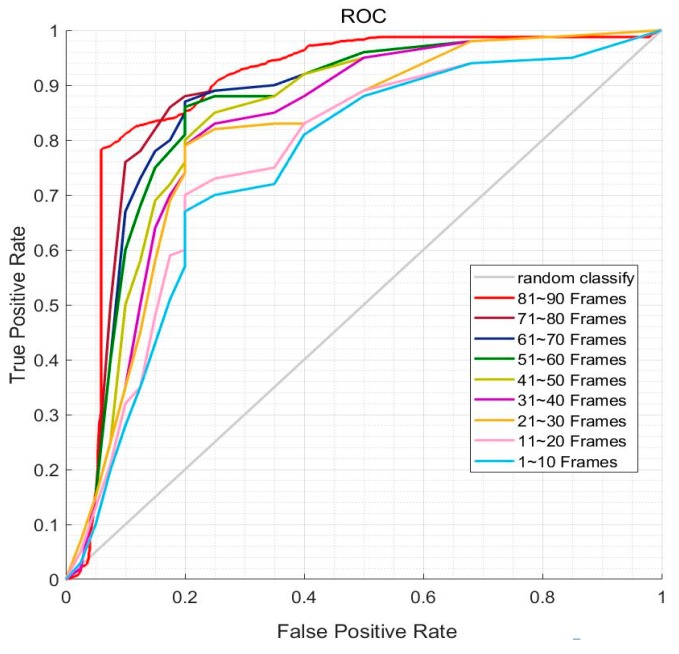
Receiver operating characteristic (ROC) curve of the BP neural network.

We chose 100 frames from a driving video and retrained the BP neural network for each 10 frames and tested the neural network with the next 10 frames. The neural network’s receiver operating characteristic (ROC) curve is shown in Figure 11. There are nine ROC curves in Figure 11, representing each 10-frame training of the neural network. With training, the neural network performance got better. More specifically, the classification performances of the BP neural network for the next 10 frames are listed in Table 2. The results illustrate that the neural network’s accuracy is equal to 0.830 at the beginning and after nine retraining frames, the accuracy increase to 0.889, which verifies that the neural network is adaptable for the current detection scenario.

In order to test the degree of improvement of the BP neural network applied to removing noise, we compared the proposed method with a hybrid median filter [47] and the regional noise removing method [48] in four different types of testing scenarios, which are listed in Table 3, including a scenario with normal illumination and good pavement; one with intense illumination and shadow interruption; another with normal illumination and a sign-on-the-ground interruption; and finally, one with poor illumination and vehicle interference. The fitting method is by the traditional RANSAC algorithm.

As shown in Table 4, in the normal illumination and good pavement scenario video, the performance of the proposed method is similar to hybrid median filter and regional noise removing, but the proposed method is better than hybrid median filter and regional noise removing in abnormal illumination and bad pavement conditions. Furthermore, the proposed method achieves a more stable and better performance in different scenarios. This experiment aims to verify the effectiveness of the proposed de-noising method, as the general fitting method, the traditional RANSAC algorithm, is applied to fit ridge-feature points to generate results in Table 4, the results in bold means the best performance in the corresponding scenarios.

### 4.3. Regional G-RANSAC Fitting Method Verification

The experimental parameters are shown as follows: scale coefficient α=1.2, fitting point number n=3, inverse transformation sampling number k=100, RANSAC iteration number 60, SGD step size λ=0.01, SGD iterations t=200 and the number of examples to use for calculating sub-gradient l=10.

To compare with Hough transform and the traditional RANSAC algorithm, we tested the proposed method in four different types of testing scenarios: normal illumination and good pavement; intense illumination and shadow interruption; normal illumination and a sign-on-the-ground interruption; and finally, one with poor illumination and vehicle interference (as listed in Table 5). The proposed de-noising method described in Section 3.3 is applied to experiment in Table 5, the results in bold means the best performance in the corresponding scenarios.

The proposed method achieved 99.02%, 96.92%, 96.65%, and 91.61% TPR in the four different testing scenarios, respectively. In addition, for the LDI, the proposed method achieved 20.55% and 26.48% more than the Hough transform and traditional RANSAC in normal illumination and good pavement conditions; 46.41% and 35.66% more in intense illumination and shadow interruption scenarios; 68.51% and 13.80% more in normal illumination and sign on the ground interruption scenarios; and 74.78% and 33.16% more in poor illumination and vehicle interferance scenario.

**Figure 12:**
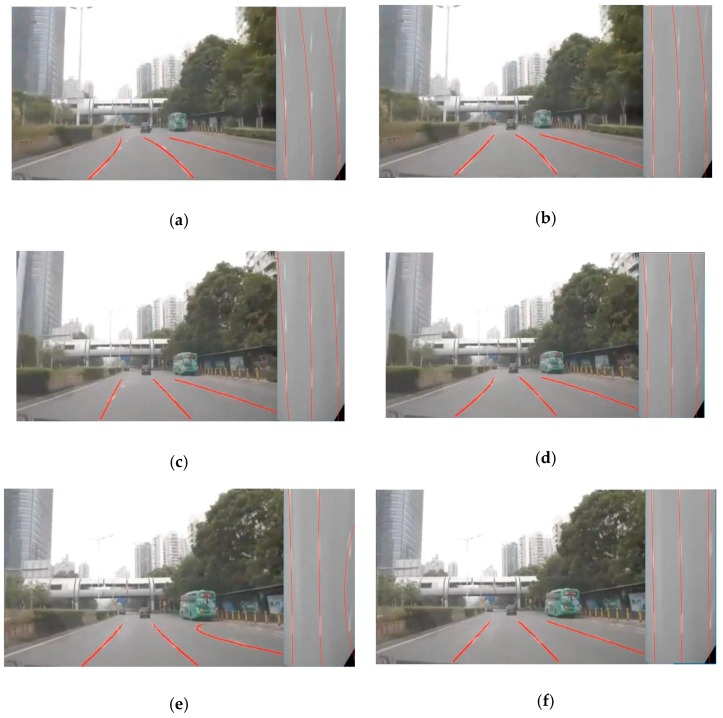
The comparative result of traditional RANSAC and regional G-RANSAC. (**a**,**c**,**e**) fitting results of the traditional RANSAC, (**b**,**d**,**f**) fitting results of the regional G-RANSAC.

In Figure 12, the comparative results of traditional RANSAC and regional G-RANSAC are shown. Figure 12a,c,e show the fitting by traditional RANSAC algorithm. Figure 12b,d,f is fitting by regional G-RANSAC. Shown in Figure 12a–d, the traditional RANSAC algorithm selects fitting points from the whole ridges’ point feature map, which increases the probability of selecting noise points. We proposed selecting fitting points from three divided areas to improve the fitting effectiveness. Shown in Figure 12e,f the traditional RANSAC algorithm missed the feature point, hence the fitting effect in Figure 12e is poor, but the proposed method considers the Gaussian distribution of feature points based on a ridges’ confidence, thus improved the robustness of lane fitting.

### 4.4. Lane Detection Frame Verifying Experience

Here, the proposed lane detection frame tests are shown. We chose recent lane detection methods to verify the effectiveness of the proposed lane detection method. Comparative methods are listed in Table 6. Testing scenario videos are the same video as above, including scenarios with normal illumination and good pavement; intense illumination and shadow interruption; normal illumination and a sign-on-the-ground interruption; and finally, one with poor illumination and vehicle interference.

As shown in Table 7 (the results in bold means the best performance in the corresponding scenarios), the proposed method has better performances than method 1 in four different types of testing scenario videos; the improvement is significant in the testing scenario of occlusion interruption, including sign interruption and vehicle interruption. The method 1 lane fitting by Hough transform, achieved 98.13% TPR and 64.10 LDI in normal illumination and good pavement condition testing scenarios, but 82.75% TPR 30.32 LDI and 85.14% TPR 33.17 LDI in sign interruption and vehicle interference scenarios, respectively. The results indicate that Hough transform is not good at the occlusion interruption scenario.

Method 3, based on vehicle trajectories, performed badly: 50.64%, 48.81%, 50.37% and 47.32% TPR; and 30.94, 21.96, 29.40 and 27.16 LDI in the four different types of testing scenarios, respectively. Additionally, method 3 fails to detect lane-division-lines in some situations; for example, when there is no preceding vehicle or the preceding vehicle changes lanes. However, compared with scenario 1, method 3 only reduced TPR 0.27% in scenario 3, while the proposed method reduced it 2.37%. The reason is that method 3 focuses on vehicle trajectories rather than lane-division line, so the fitting result is not affected by the sign marking on the ground.

The proposed method has satisfactory performances in four different types of testing scenarios, achieving 99.02%, 96.92%, 96.65% and 91.61% TPR; and 66.16, 54.85, 55.98 and 52.61 LDI in the four different types of testing scenarios, respectively. The results show that the proposed method is effective in challenging scenarios.

**Figure 13:**
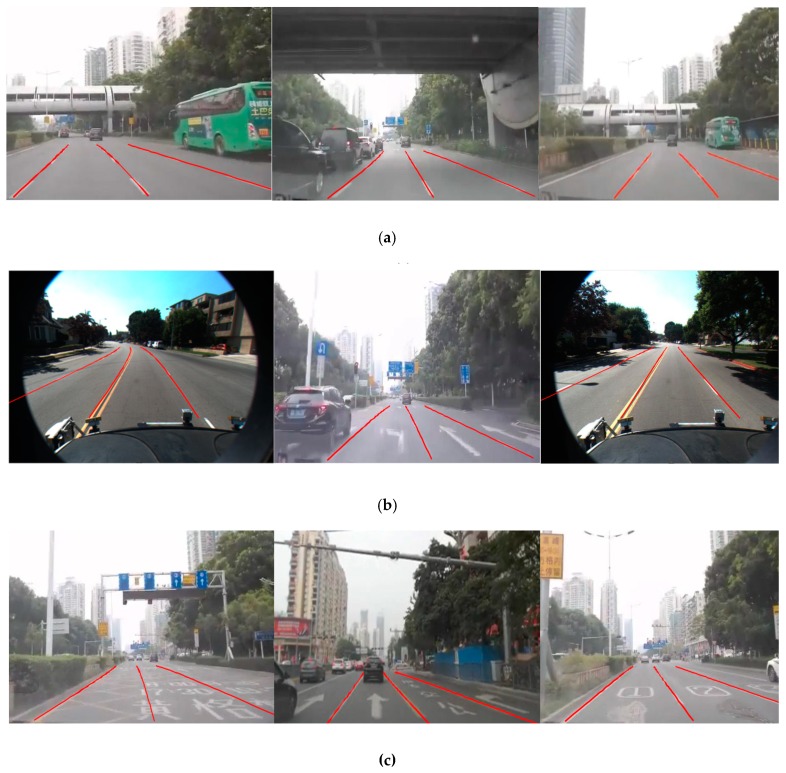

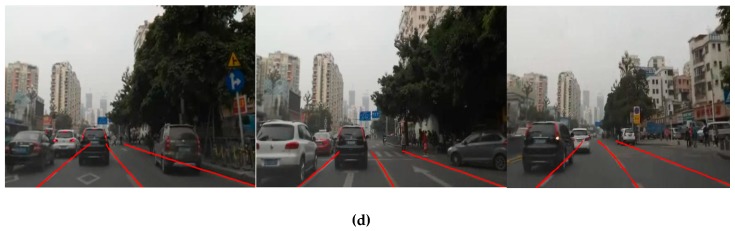
The proposed method detects the lane in four different types of scenarios. (**a**) Scenario 1 with normal illumination and good pavement. (**b**) Scenario 2 with intense illumination and shadow interruption. (**c**) Scenario 3 with normal illumination and sign-on-the-ground interruption, and (**d**) scenario 4 with poor illumination and vehicle interference.

In Figure 13a, because of good lighting conditions and road conditions, there are few noise points in the ridge-feature map, resulting in good detection. In Figure 13b, the testing scenario has shadow interruption due to strong lighting, whereas the proposed method can remove shadow noise points and show good lane-division line fitting. In Figure 13c, the sign on the ground is the main interference, but the lane-division line model and BP neural network has the capability to discriminate between ridge pixel points and sign pixel points, which results in a good git. In Figure 13d, the scenario has poor illumination, vehicle interfere generates a lot of noise points and it lacks key ridge points; however, with the advantage of the G-RANSAC algorithm, the proposed method can fit the lane-division line with a small number of ridge points.

**Figure 14:**
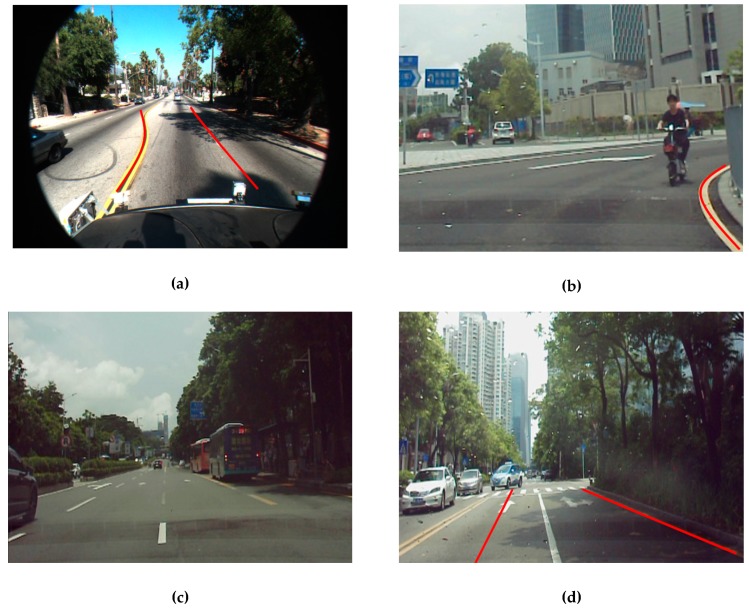
Incorrect detection with the proposed method. (**a**) The left lane-division line is not a good fit. (**b**) Detection failure of the left lane division. (**c**) Detection failure of lane division. (**d**) The left predicted lane division does not match the left, real lane-division line.

Herein, the drawbacks of the proposed method are described. First, as the proposed method fitting model, Parabola, cannot provide perfect fitting when the lane-division line bends continuously, shown in Figure 14a. Second, the proposed method fails to detect lane-division-lines when its abscissa crosses three ranges (25, 60) and (150, 190) and (285, 300) at same time, shown in Figure 14b. Third, the proposed method fails to detect lane-division-lines when the vehicle itself changes lane, shown in Figure 14c,d.

## 5. Conclusions

In this paper, we proposed a lane-division-lines detection method based on ridge detector and regional G-RANSAC. The main innovation is summarized as: First, we removed noise points by an adaptable neural network. The experimental results verified that the adaptable neural network achieves better detection performance than a hybrid median filter and regional noise removing, in challenging scenarios. Secondly, we improved the traditional RANSAC by considering the confidence levels of pending fitting points. The experimental results indicate that the regional G-RANSAC achieves better detection performance in TPR and LDI compared to traditional RANSAC and Hough transform in different scenarios. Last, we compared the whole proposed method with other lane detection methods on four types of testing scenario videos, including a scenario with normal illumination and good pavement; one with intense illumination and shadow interruption; another with normal illumination and a sign-on-the-ground interruption; and finally, one with poor illumination and vehicle interference. The experimental results show, regardless of normal or challenging scenarios, the proposed method achieves 0.91%, 9.85%, 10.57% and 7.60% improvements in TPR; and 3.21%, 43.47%, 84.63% and 58.61% improvements in LDI in the four different types of testing scenarios compared to the other lane detection methods, especially in the sign and vehicle interference scenario. Since the proposed method cannot adaptively separate the lane-division line regions on the abscissa, the lane-division line cannot be well fitted in the case where the lane dividing line bends continuously and the vehicle changes lanes. Variable region bounds are an effective method to solve the problem [49], which will be studied in the future.

## Figures and Tables

**Table 1:** Comparison experiment about the operating speed of the traditional method and the proposed method.

Testing Scenario Video	Method [43] (Frame/s)	Proposed Method (Frame/s)
Scenario 1	32	82
Scenario 2	30	81
Scenario 3	31	79
Scenario 4	34	85

**Table 2:** Result of the BP neural network for different frames.

Train Dataset	Test Dataset	Accuracy	mAP
1–10 frames	11–20 frames	0.830	0.753
11–20 frames	21–30 frames	0.855	0.776
21–30 frames	31–40 frames	0.861	0.802
31–40 frames	41–50 frames	0.860	0.816
41–50 frames	51–60 frames	0.877	0.832
51–60 frames	61–70 frames	0.882	0.847
61–70 frames	71–80 frames	0.881	0.854
71–80 frames	81–90 frames	0.886	0.865
81–90 frames	91–100 frames	0.889	0.883

**Table 3:** Different testing video scenarios.

Testing Scenario Video	Illumination Condition	Pavement Condition	Frame Number
Scenario 1	normal	good	2327
Scenario 2	intense	shadow interrupt	2139
Scenario 3	normal	road sign interrupt	2231
Scenario 4	poor	vehicle interrupt	2391

**Table 4:** Comparative results of noise removing methods.

Testing Scenario Video	Noise Removing Methods	TPR (%)	LDI
Scenario 1	Hybrid median filter	93.81	42.88
	Regional noise removing	94.92	50.19
	Proposed method	**95.13**	**52.31**
Scenario 2	Hybrid median filter	83.91	32.71
	Regional noise removing	86.32	35.45
	Proposed method	**92.21**	**40.16**
Scenario 3	Hybrid median filter	85.03	33.16
	Regional noise removing	86.12	37.52
	Proposed method	**91.32**	**49.19**
Scenario 4	Hybrid median filter	85.91	32.02
	Regional noise removing	84.23	35.54
	Proposed method	**90.43**	**39.51**

**Table 5:** Comparative results of fitting methods in different test scenarios.

Testing Scenario Video	Fitting Method	TPR (%)	LDI
Scenario 1	Hough transform	98.87	54.88
	Traditional RANSAC	95.13	52.31
	Proposed method	**99.02**	**66.16**
Scenario 2	Hough transform	88.71	37.21
	Traditional RANSAC	92.21	40.16
	Proposed method	**96.92**	**54.48**
Scenario 3	Hough transform	85.67	33.22
	Traditional RANSAC	91.32	49.19
	Proposed method	**96.65**	**55.98**
Scenario 4	Hough transform	83.21	30.10
	Traditional RANSAC	90.43	39.51
	Proposed method	**91.61**	**52.61**

**Table 6:** Comparison of lane detection methods.

Lane Detection	Preprocess	Feature Extraction	Lane Model	Fitting Method
Method 1 [4]	Median filter	Sobel filter	Straight line	Hough transform
Method 2 [9]	ROI selection based on vanishing point	Canny filter	Straight line	Least square
Method 3 [38]	Gray image	Vehicle trajectories	Straight line	Least square
Proposed method	Inverse perspective transformation	Ridge detector	Parabola	Regional G-RANSAC

**Table 7:** Comparative results of lane detection methods.

Testing Scenario Video	Lane Detection Method	TPR (%)	LDI
Scenario 1	Method 1	98.13	64.10
	Method 2	94.54	49.28
	Method 3	50.64	30.94
	Proposed method	**99.02**	**66.16**
Scenario 2	Method 1	88.23	38.23
	Method 2	87.75	31.18
	Method 3	48.81	21.96
	Proposed method	**96.92**	**54.85**
Scenario 3	Method 1	82.75	30.32
	Method 2	87.41	34.19
	Method 3	50.37	29.40
	Proposed method	**96.65**	**55.98**
Scenario 4	Method 1	85.14	33.17
	Method 2	82.66	29.90
	Method 3	47.32	27.16
	Proposed method	**91.61**	**52.61**
